# Bayesian Phylogeographic Inference Suggests Japan as the Center for the Origin and Dissemination of Rice Stripe Virus

**DOI:** 10.3390/v14112547

**Published:** 2022-11-17

**Authors:** Kangcheng Wu, Yunyue Yang, Wenwen Zhang, Xiaofeng Jiang, Weijian Zhuang, Fangluan Gao, Zhenguo Du

**Affiliations:** 1 Fujian Key Laboratory of Plant Virology, Institute of Plant Virology, Fujian Agriculture and Forestry University, Fuzhou 350002, China; 2 Ministerial and Provincial Joint Innovation Centre for Safety Production of Cross-Strait Crops, Fujian Agriculture and Forestry University, Fuzhou 350002, China

**Keywords:** rice stripe virus, phylogeography, ancestral location, virus dispersal

## Abstract

Rice stripe virus (RSV) is one of the most important viral pathogens of rice in East Asia. The origin and dispersal of RSV remain poorly understood, but an emerging hypothesis suggests that: (i) RSV originates from Yunnan, a southwest province of China; and (ii) some places of eastern China have acted as a center for the international dissemination of RSV. This hypothesis, however, has never been tested rigorously. Using a data set comprising more than 200 time-stamped coat protein gene sequences of RSV from Japan, China and South Korea, we reconstructed the phylogeographic history of RSV with Bayesian phylogeographic inference. Unexpectedly, the results did not support the abovementioned hypothesis. Instead, they suggested that RSV originates from Japan and Japan has been the major center for the dissemination of RSV in the past decades. Based on these data and the temporal dynamics of RSV reported recently by another group, we proposed a new hypothesis to explain the origin and dispersal of RSV. This new hypothesis may be valuable for further studies aiming to clarify the epidemiology of RSV. It may also be useful in designing management strategies against this devastating virus.

## 1. Introduction

*Rice stripe virus* (RSV) is a plant-infecting bunyavirus belonging to the genus *Tenuivirus* of the family *Phenuiviridae* [1]. It has a genome consisting of four single-stranded RNA segments that are named RNA1, RNA2, RNA3 and RNA4, respectively, according to the decreasing order of their size. RNA1 contains an open reading frame (ORF) on the complementary-sense strand. RNA2–RNA4 each contain two ORFs, one on the 5′-half of the complementary-sense strand and the other on the 5′-half of the virus-sense strand. Within host cells, RNA1–RNA4 are each encapsidated by multiple copies of the viral nucleocapsid protein, forming four distinct ribonucleoprotein complexes varying in size. Unlike most other bunyaviruses, RSV does not form enveloped virions throughout its life cycle [1,2]. RSV is transmitted in a circulative and propagative manner by the small brown planthopper *Laodelphax striatellus* Fallén. It is the causal agent of a devastating disease named rice stripe disease, which occurs widely in many east Asian countries including Japan, China and South Korea [2,3,4,5]. Rice plants affected by this disease develop chlorotic spots on their leaves. The spots become more numerous with time and eventually fuse to form chlorotic stripes. If the infection takes place in the early growth stage of rice, the affected plants may die prematurely [3,4].

The origin and dispersal of RSV remain poorly understood, but a hypothesis is emerging from studies examining the population structure of this virus [6,7,8,9]. Overall, these studies revealed that RSV falls into two major clades. Whereas clade I is distributed across China, Japan and South Korea, clade II is restricted to Yunnan, a southwest province of China. This suggests that RSV may originate from Yunnan. Incidentally, one RSV lineage was transported to eastern China before being further dispersed to Japan and South Korea. In line with this hypothesis, mass immigrations of *L. striatellus* were recently detected in Japan and South Korea. In both cases, the source of the immigrants was tracked to Jiangsu, an eastern province of China [10,11,12]. This suggests that the barrier for the international dispersal of RSV is easily circumventable provided that RSV can reach some places of eastern China.

The past decade has witnessed great advances in a discipline called phylodynamics. With these advances, it has become possible to statistically infer the epidemiological processes of a virus [13,14,15]. He and colleagues first analyzed the temporal dynamics of RSV. By doing this, they obtained a time-scaled maximum clade credibility (MCC) tree of RSV [16]. Interestingly, this revealed two candidates for the origin of RSV, namely Yunnan of southwest China and Japan. However, probably because the authors made no attempt to infer the root location of their MCC tree [17], the statistical supports for either candidate were not reported [15]. As an extension of this work, we inferred the origin location of RSV with a recently developed phylographic method named Bayesian structured coalescent approximation [18]. Based on this, we also inferred the migration patterns of RSV. Unexpectedly, our results strongly suggest that RSV originates from Japan and Japan has been the major center for the dissemination of RSV in the past decades.

## 2. Materials and Methods

**Figure 1:**
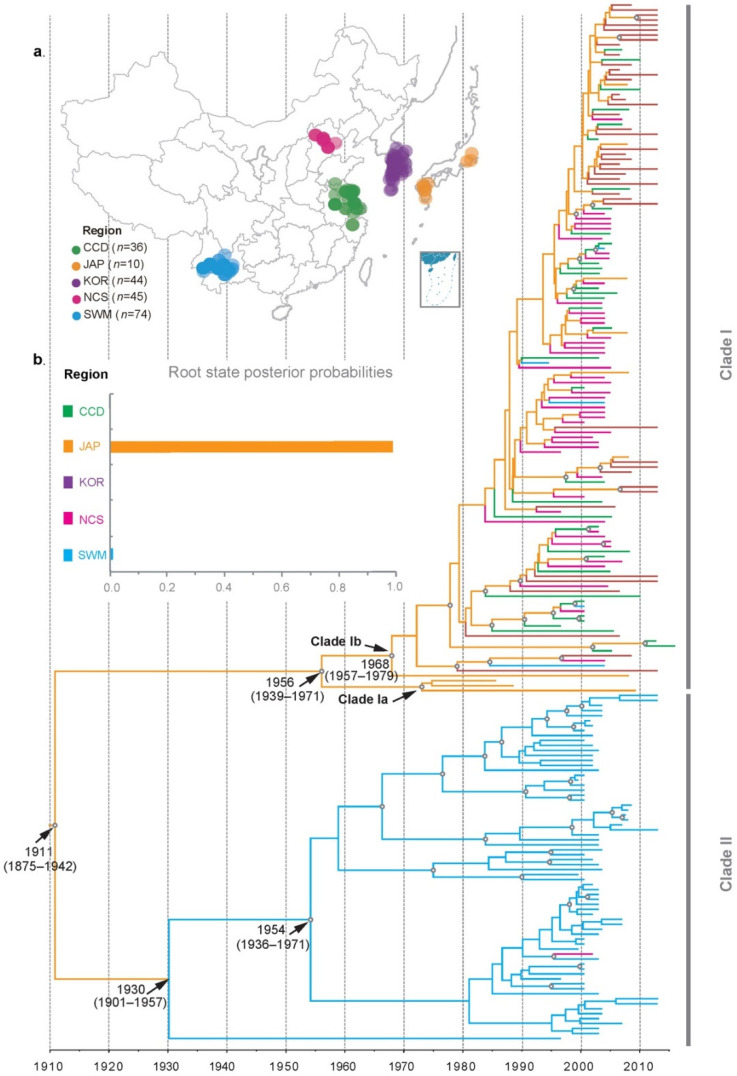
The phylogeography of rice stripe virus (RSV). (**a**) A map showing the geographical distribution of RSV. (**b**) Time-scaled maximum clade credibility tree inferred from CP sequences of rice strip virus. Branch colors represent most probable inferred location states, as indicated by the color key. The empty circles are sized in proportion to node posterior probabilities (only those with a value >0.95 were shown). The inset panel is the root state posterior probability estimated for each region. Abbreviations: the central China double cropping (CCD) zone, the southwest mix cropping (SWM) zone, the north China single cropping (NCS) zone, Japan (JAP) and South Korea (KOR).

The coat protein (CP) gene sequences of 209 RSV isolates were obtained from GenBank (Supplemental Table S1). These RSV isolates were sampled between 1986 and 2016 from China (*n* = 155), South Korea (*n* = 44) or Japan (*n* = 10). The rice in China was grown in six distinct agro-ecological zones, namely the southeast China double cropping (SCD) zone, the central China double cropping (CCD) zone, the southwest mix cropping (SWM) zone, the north China single cropping (NCS) zone, the northeast China single cropping (NES) zone and the northwest single cropping (NWS) zone. The RSV isolates used in this study come from three of them, namely the CCD (*n* = 36), the NCS (*n*= 45) and the SWM (*n* = 74, Figure 1a) zones. All SWM zone RSV isolates come from Yunnan Province. 

A codon-based sequence alignment was performed using the MAFFT [19] integrated in PhyloSuite 1.21 [20]. The final sequence alignment was composed of 966 nucleotides excluding the stop codons. Seven algorithms (RDP, GENECONV, BOOTSCAN, MAXCHI, CHIMAERA, SISCAN and 3SEQ) implemented in RDP 4.95 packages [21] were employed to detect recombinants in the data set. Recombinants detected by at least four of the seven algorithms with *p* <10^−6^ were considered to be true recombinants. According to these criteria, no evidence of recombination was detected in the data and therefore we included the complete data set for our subsequent analyses.

A likelihood mapping analysis was performed to investigate the phylogenetic signal in the aligned sequences using the quartet puzzling algorithm [22]. For this analysis, 5500 quartets of sequences were randomly selected and analyzed using the program IQ-TREE 1.6.6 [23] under the best-fit substitution model GTR+F+*Г*_4_, which was selected using ModelFinder [24] implemented in PhyloSuite. We found that 73.2% of all quartets were fully resolved triangles (unresolved quartets = 20.3% < 33.0%, Figure S1), indicating a strong tree-like phylogenetic signal in our data set for reliable phylogenetic inference.

To explore the evolutionary history of RSV, we employed the Bayesian coalescent approaches implemented in BEAST 2.5.2 [25] using the GTR+F+*Г*_4_ substitution model, as selected above. We combined two molecular clock models (strict and uncorrelated lognormal relaxed) with three different demographic models (the constant-size coalescent, approximate structured coalescent and Bayesian skyline coalescent tree priors) to choose the best combination model. A structured coalescent [26] tree prior and strict clock models provided the best fit to the data according to marginal likelihoods estimated by path sampling (Supplemental Table S2). Default priors were used for all parameters. 

The sampling dates of each isolate were used to calibrate the molecular clock. To assess the time structure in the data set, we first regressed root-to-tip distances against date of sampling using TempEst 1.5 [27] and further confirmed the presence of the temporal signal using a clustered date-randomization test recommended by Duchêne [28]. However, no temporal structure was identified in our data set using either of the two methods and thus a uniform prior of 2.99 × 10^−4^ − 5.73 × 10^−4^ substitutions/site/year was specified for the absolute substitution rate of the CP gene, estimated from a previous study [16]. All Bayesian analyses were performed with three independent Markov chains run for 100 million steps, and samples were collected every 10,000 steps. The resulting convergence, accepting only values higher than 200 for all the parameters, was examined using Tracer 1.71 [29]. After removing the top 10% samples (burn-in), the maximum clade credibility (MCC) tree was summarized using TreeAnnotator from the BEAST package and visualized with FigTree 1.4.4 (http://tree.bio.ed.ac.uk/software/figtree/, accessed on 15 November 2022).

To infer the migration history of RSV through time, we employed a reference script by Brynildsrud et al. [30] (https://github.com/admiralenola/globall4scripts, accessed on 22 October 2022) to analyze the inferred load and direction of the MCC tree from the MASCOT runs. In this analysis, migration events were set to occur on nodes, although they in reality could occur at any points along the branch. This introduces a slight bias towards the inflated ages of the migration events.

## 3. Results

### 3.1. The Phylogeography of Rice Stripe Virus

In agreement with the report of He et al. [16], our time-scaled MCC tree resolved RSV into two major clades whose divergence occurred around 1911 (95% credibility interval 1875–1942, Figure 1b). Whereas clade I isolates have a wide geographical distribution, those of clade II are all from the SWM zone of China (Yunnan Province, China), with only one exception. The time of the most recent common ancestor (TMRCA) of clade I was dated to 1956 (95% credibility interval 1939–1971). However, the majority of clade I isolates seemed to share a more recent ancestor that diverged in 1968 (95% credibility interval 1957–1979). The only three RSV isolates that were not descendants of this ancestor came from Japan. They formed a subclade separable from all other clade I isolates. For the ease of presentation, we hereafter call this subclade clade Ia and the subclade formed by the remaining clade I isolates clade Ib (Figure 1b). A similar observation was made from clade II isolates: whereas their TMRCA was dated to 1930 (95% credibility interval 1901–1957), all but one of them were descendants of a more recent common ancestor that diverged around 1954 (95% credibility interval 1936–1971).

The ancestry state of RSV was determined by the Bayesian structured coalescent approximation approach [18]. Unexpectedly, the spatial origin of RSV was placed in Japan (root posterior probability = 0.99). Clade Ia contained two RSV isolates that are much older than all other isolates analyzed in this study (Figure 1b). To test whether these two isolates influence ancestry placement, we deliberately excluded them from the analysis. The ancestry location of RSV was not influenced (Supplemental Figure S2). A similar observation was made when all three RSV isolates of clade Ia were excluded (Supplemental Figure S3). 

We also tried to exclude all Japanese or all Japanese and South Korea isolates before conducting the same analysis. When all Japanese were excluded, the spatial origin of RSV was placed in South Korea (Supplemental Figure S4). When all Japanese and South Korea isolates were excluded, the spatial origin of RSV was placed in the CCD of China (Supplemental Figure S5). Thus, it seems that a greater genetic diversity of RSV in Yunnan cannot be used as evidence to locate the origin of RSV to this place.

### 3.2. The Dispersal Patterns of Rice Stripe Virus

**Figure 2:**
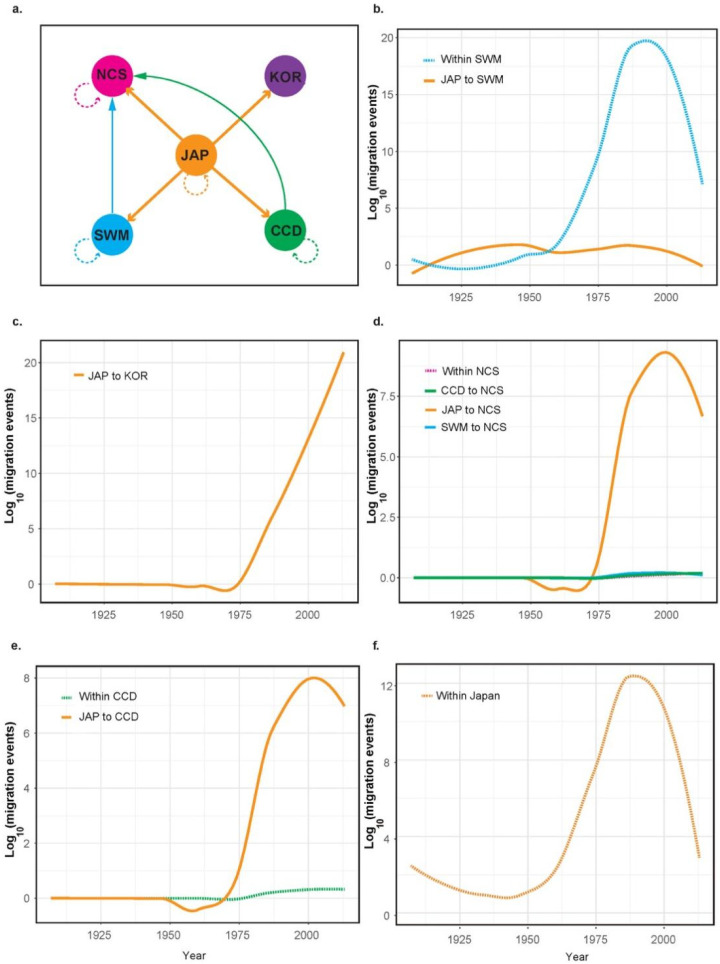
Migrations of RSV. (**a**) An overview of RSV migrations occurring among or within countries/zones. (**b**–**f**) The plots showing migration events over time with each country/zone as the sink. The *x*-axes are measured in calendar years and the *y*-axes indicate migration events on log_10_ scale. Abbreviations: the central China double cropping (CCD) zone, the southwest mix cropping (SWM) zone, the north China single cropping (NCS) zone, Japan (JAP) and South Korea (KOR).

With a statistically supported spatial origin available, we next sought to examine epidemiologically significant lineage migrations of RSV. Consistent with the origin location of RSV, Japan was found to be a major source for RSV emigrations (Figure 2a). The first wave of RSV emigrations from Japan started from the 1920s and had Yunnan (the SWM zone of China) as the sink (Figure 2b). Overall, the rates of the Japan to Yunnan RSV migrations were very low. The second wave of RSV emigrations from Japan started from the mid-1970s, with eastern China (the CCD and NCS zones) and South Korea as the sinks (Figure 2c,d). The rates of Japan to eastern China migrations began to drop in the early 2000s, but those of Japan to South Korea kept increasing. As well as Japan, RSV emigrations were also seen from the CCD and SWM zones of China, both with the NCS zone of China as the sink. The rates of these migrations, however, were much lower than those with Japan as the source (Figure 2c,d). In all the cases examined, except South Korea, within-country/zone migrations of RSV were detectable. However, high rates of within-country/zone migrations were observed only in Yunnan and Japan (Figure 2b,e).

## 4. Discussion

The most prevailing hypothesis about the origin and dispersal of RSV states that RSV originates from Yunnan Province, China. It also predicts that some places in eastern China have acted as a center for the international dissemination of RSV. Apparently, the results obtained from this study did not support this hypothesis. First, the results of ancestry location did not support Yunnan as the origin of RSV. The statistics excluding Yunnan as the origin of RSV are robust, as suggested by our analyses in which RSV sequences from Japan and South Korea were deliberately excluded. Secondly, the dispersal patterns of RSV determined in this study did not support the presence of an RSV dissemination center in eastern China. Although this does not deny the occurrence of occasional overseas emigrations of RSV from eastern China, it strongly suggests that these emigrations have little effect on the population structure of RSV in Japan/South Korea, at least in the past decades.

Together with those of He et al. [16], our results point to a new hypothesis. According to this hypothesis, RSV emerged from Japan before the late 1900s. It diverged there at a year around 1911, giving rise to the ancestor of the clade I and II RSV found nowadays. Shortly thereafter, RSV began to perform long-distance movements, most probably facilitated by *L. striatellus*. In eastern China/South Korea, immigrations of RSV occurred frequently. In Yunnan, however, immigrations of RSV occurred only occasionally because it is too far away from Japan. It is reasonable to assume that the ancestor of clade I and II RSV coexisted in Japan for years and they had equal chances to be exported from Japan in this period.

During dissemination, evolution forces continually acted to shape the population structure of RSV. In Japan, these forces resulted in a rapid extinction of clade II. In Yunnan, by contrast, these forces facilitated the flourishment of clade II. The nature of these evolution forces is unclear. However, the ability of *L. striatellus* to transmit RSV varies greatly depending on their genetic background [2,3]. This suggests that *L. striatellus* may impose a strong selection pressure on RSV. In addition, because introductions of clade I have never been stopped, the likelihood that genetic drift has played a major role in the flourishment of only clade II in Yunnan can be excluded. 

The ecological conditions in eastern China/South Korea might have been unfavorable for both clades of RSV until the late 1960s. As a result, RSV was not established in these two sites despite decades of frequent introductions. From the late 1960s onwards, however, the situation changed so that the introduced RSV began to flourish. There are two explanations for the change. First, RSV had evolved to be more adaptive. Secondly, the ecological conditions in eastern China and South Korea might have changed. Considering the fact that RSV experienced an epidemic in Japan in the 1960s, we favor the first explanation [3,4]. However, the idea that ecological conditions in eastern China and South Korea have changed since the late 1960s is also plausible.

Many more studies are needed to substantiate the new hypothesis. For example, this hypothesis predicts frequent Japan to China/South Korea movements of *L. striatellus*. Such movements have not been demonstrated experimentally, although Japan to China migrations of *L. striatellus* have been proposed recently based on genetic analysis of *L. striatellus* [31]. In addition, the number of RSV sequences from Japan is very small (*n* = 10). The number from South Korea is bigger (*n* = 44), but most sequences are collected from only a few years. Although the methods used here are declared to be robust to sample heterogeneity, further studies are needed to be more conservative [18]. Nevertheless, it is notable that the new hypothesis is more consistent with historical records than the previous one: rice stripe disease was first noted in Japan. By the early 1960s when it was noted in eastern China and South Korea, this disease had been a well-known endemic in Japan for more than 60 years [3,4]. At least in eastern China, where rice has been a major source for livelihood and revenue for centuries, it is difficult to envisage a scenario in which rice stripe disease had occurred without being noticed for such a long time. 

## 5. Conclusions

In summary, with the help of several recently developed phylogeographic methods, we obtained some fresh insights into the origin and dispersal of RSV. These insights may be valuable in clarifying the epidemiology of RSV, as well as in designing management strategies against this devastating virus. Our report may also serve as a case study suggesting that cautions are needed in determining the origin of a virus based solely on the topology of its phylogeny. 

## Data Availability

All data used in this study are publicly available on NCBI. A list of the accession numbers used is found in Supplementary Table S1.

## Supplementary Materials

The following supporting information can be downloaded at: https://www.mdpi.com/article/10.3390/v14112547/s1, Figure S1: Likelihood mapping analysis for the CP gene data set; Figure S2: Maximum clade credibility tree constructed with the data set in which the two eldest RSV sequences from Japan were excluded; Figure S3: Maximum clade credibility tree constructed with the data set in which the three clade Ia RSV sequences from Japan were excluded; Figure S4: Maximum clade credibility tree constructed with the data set in which all the RSV sequences from Japan were excluded; Figure S5: Maximum clade credibility tree constructed with the data set in which all the RSV sequences from Japan and South Korea were excluded; Table S1: Rice stripe virus isolates analyzed in this study title; Table S2: Marginal likelihoods of different combinations of clock model and tree prior.
