# Supplementary material for: Bayesian Phylogeographic Inference Suggests Japan as the Center for the Origin and Dissemination of Rice Stripe Virus
Source: Viruses. 2022 Nov 17;14(11):2547. doi: 10.3390/v14112547 (PMC9698939; doi:10.3390/v14112547)
Supplement: Supplementary file 1 [file viruses-14-02547-s001.zip › Table S1.pdf]

Table S1 Rice stripe virus isolates used in this study

No.	Isolate	Country	Region	Host	Collection date	ACCESSION
1	ABB07	China	CCD	*Oryza sativa*	2007-7-1	EU931524
2	JS.FS.04	China	CCD	*Oryza sativa*	2004	AY973309
3	AHF1.04	China	CCD	*Oryza sativa*	2004-7-1	DQ289589
4	AHF2.04	China	CCD	*Oryza sativa*	2004-7-1	DQ289590
5	AHF3.04	China	CCD	*Oryza sativa*	2004-7-1	DQ289591
6	AHF1.05	China	CCD	*Oryza sativa*	2005-7-1	DQ299153
7	AHF2.05	China	CCD	*Oryza sativa*	2005-7-1	DQ299154
8	HZ	China	CCD	*Oryza sativa*	2001	AF508865
9	HZ	China	CCD	*Oryza sativa*	2001	AY286108
10	JS.HZ.03	China	CCD	*Oryza sativa*	2003-7-1	AY597379
11	LS.JSHZ03	China	CCD	*Oryza sativa*	2003-9-3	EF198685
12	HA	China	CCD	*Oryza sativa*	2001	AY286109
13	JD	China	CCD	*Oryza sativa*	1997	AF220104
14	IOZ	China	CCD	*Oryza sativa*	2016-6-20	MF287953
15	JS.YH.04	China	CCD	*Oryza sativa*	2004	AY973308
16	JS.YM	China	CCD	*Triticum aestivum*	2006-8-31	AM397834
17	JSu1	China	CCD	*Oryza sativa*	2013-4-3	KP083238
18	CN.JSCX08	China	CCD	*Laodelphax striatellus*	2008-10-1	AB622862
19	CN.JSYX08	China	CCD	*Laodelphax striatellus*	2008-10-1	AB622863
20	DZ	China	CCD	*Oryza sativa*	2001	AY284864
21	JY	China	CCD	*Oryza sativa*	2006	EF141329
22	JS.JH.04	China	CCD	*Oryza sativa*	2004	AY973310
23	LS.JSJJ03	China	CCD	*Oryza sativa*	2003-7-26	EF198682
24	LS.JSJJ054	China	CCD	*Oryza sativa*	2005-9-30	EF198681
25	LS.JSJJ051	China	CCD	*Oryza sativa*	2005-9-30	EF198691
26	LS.JSJJ052	China	CCD	*Oryza sativa*	2005-9-30	EF198693
27	LS.JSPX03	China	CCD	*Oryza sativa*	2003-6-14	EF198699
28	JiangY10	China	CCD	*Oryza sativa*	2010-7-25	JQ927422
29	SuZ10	China	CCD	*Oryza sativa*	2010-7-25	JQ927425
30	JYC07	China	CCD	*Oryza sativa*	2007-7-1	EU931516
31	LS.JSYD03	China	CCD	*Oryza sativa*	2003-7-21	EF198688
32	LS.JSYD051	China	CCD	*Oryza sativa*	2005-10-5	EF198689
33	LS.JSYD052	China	CCD	*Oryza sativa*	2005-10-5	EF198692
34	Zhejiang	China	CCD	*Oryza sativa*	2003-7-1	DQ333944
35	HuZ10	China	CCD	*Oryza sativa*	2010-7-25	JQ927421
36	CN.ZAHZ08	China	CCD	*Laodelphax striatellus*	2008-9-1	AB622861
37	JP.FKYM08	Japan	JAP	*Laodelphax striatellus*	2008-6-1	AB622859
38	JP.KGMS08	Japan	JAP	*Laodelphax striatellus*	2008-6-1	AB622856
39	JP.KMAK08	Japan	JAP	*Laodelphax striatellus*	2008-6-1	AB622857
40	JP.KMKS08	Japan	JAP	*Laodelphax striatellus*	2008-5-1	AB622858
41	JP.NSTG08	Japan	JAP	*Laodelphax striatellus*	2008-8-1	AB622860

42	JP.R.NSTG08	Japan	JAP	*Oryza sativa*	2008-8-1	AB622864
43	JP.R.NSHO08	Japan	JAP	*Oryza sativa*	2008-8-1	AB622865
44	Japan.M	Japan	JAP	*Oryza sativa*	1986	D01094
45	JP.TGOY09	Japan	JAP	*Laodelphax striatellus*	2009-10-1	AB622866
46	isolate T	Japan	JAP	*Triticum aestivum*	1989	X53563
47	AD.JJ	South Korea	KOR	*Oryza sativa*	2007	FJ602684
48	BA1.JB	South Korea	KOR	*Oryza sativa*	2007	FJ602675
49	BA2.JB	South Korea	KOR	*Oryza sativa*	2007	FJ602676
50	Cheon'an	South Korea	KOR	*Oryza sativa*	2013-7-1	KF885681
51	Cheongwon	South Korea	KOR	*Oryza sativa*	2013-7-1	KF885683
52	Cheorwon2	South Korea	KOR	*Oryza sativa*	2013-7-1	KF885685
53	CY.CN	South Korea	KOR	*Oryza sativa*	2007	FJ602682
54	SC.CN6	South Korea	KOR	*Oryza sativa*	2009	HQ343292
55	SC.CN70	South Korea	KOR	*Oryza sativa*	2009	HQ343293
56	TA.CN5	South Korea	KOR	*Oryza sativa*	2009	HQ343294
57	Dangjin	South Korea	KOR	*Oryza sativa*	2013-7-1	KF885687
58	Gangneung	South Korea	KOR	*Oryza sativa*	2013-7-1	KF885689
59	Goseong.a2	South Korea	KOR	*Oryza sativa*	2013-7-1	KF885697
60	Geochang	South Korea	KOR	*Oryza sativa*	2013-7-1	KF885691
61	Gimcheon	South Korea	KOR	*Oryza sativa*	2013-7-1	KF885693
62	Gimje	South Korea	KOR	*Oryza sativa*	2013-7-1	KF885695
63	GC.JB	South Korea	KOR	*Oryza sativa*	2007	FJ602679
64	GS.JB	South Korea	KOR	*Oryza sativa*	2007	FJ602677
65	Goseong.b	South Korea	KOR	*Oryza sativa*	2013-7-1	KF885699
66	Haenam	South Korea	KOR	*Oryza sativa*	2013-7-1	KF885701
67	Hwaseong	South Korea	KOR	*Oryza sativa*	2013-7-1	KF885703
68	IS.JB	South Korea	KOR	*Oryza sativa*	2007	FJ602678
69	Jecheon	South Korea	KOR	*Oryza sativa*	2013-7-1	KF885705
70	Jeju	South Korea	KOR	*Oryza sativa*	2013-7-1	KF885707
71	BA.JB6	South Korea	KOR	*Oryza sativa*	2009	HQ343287
72	BA.JB16	South Korea	KOR	*Oryza sativa*	2009	HQ343288
73	GS.JB16	South Korea	KOR	*Oryza sativa*	2009	HQ343289
74	JD.JN4	South Korea	KOR	*Oryza sativa*	2009	HQ343290
75	JD.JN6	South Korea	KOR	*Oryza sativa*	2009	HQ343291
76	Jincheon	South Korea	KOR	*Oryza sativa*	2013-7-1	KF885709
77	JD.JN	South Korea	KOR	*Oryza sativa*	2007	FJ602681
78	Miryang	South Korea	KOR	*Oryza sativa*	2013-7-1	KF885711
79	MA.JN	South Korea	KOR	*Oryza sativa*	2007	FJ602680
80	Naju	South Korea	KOR	*Oryza sativa*	2013-7-1	KF885713
81	Pyeongtaek	South Korea	KOR	*Oryza sativa*	2013-7-1	KF885715
82	Sacheon2	South Korea	KOR	*Oryza sativa*	2013-7-1	KF885717
83	Sangju	South Korea	KOR	*Oryza sativa*	2013-7-1	KF885719
84	SC.CN	South Korea	KOR	*Oryza sativa*	2007	FJ602683
85	Seosan	South Korea	KOR	*Oryza sativa*	2013-7-1	KF885721

86	SA.JN	South Korea	KOR	*Oryza sativa*	2008	FJ602685
87	Suwon	South Korea	KOR	*Oryza sativa*	2006-5-1	GU230170
88	Uiseong	South Korea	KOR	*Oryza sativa*	2013-7-1	KF885723
89	WD.JN	South Korea	KOR	*Oryza sativa*	2008	FJ602686
90	YG.JN	South Korea	KOR	*Oryza sativa*	2008	FJ602687
91	BJ1	China	NCS	*Oryza sativa*	2001	AY284873
92	BCP2.04	China	NCS	*Oryza sativa*	2004-7-1	DQ299151
93	BCP1.04	China	NCS	*Oryza sativa*	2004-7-1	DQ299152
94	BCP4.04	China	NCS	*Oryza sativa*	2004-7-1	DQ302798
95	ND.2002	China	NCS	*Oryza sativa*	2002-7-1	AY289045
96	HN.ND.04	China	NCS	*Oryza sativa*	2004	AY973311
97	JN1	China	NCS	*Oryza sativa*	2001	AY284872
98	SD.JN2	China	NCS	*Oryza sativa*	2005	DQ108406
99	SJN1.05	China	NCS	*Oryza sativa*	2005-8-1	DQ299155
100	SJN2.05	China	NCS	*Oryza sativa*	2005-8-1	DQ299156
101	SJN1.04	China	NCS	*Oryza sativa*	2004-8-1	DQ299167
102	SJN2.04	China	NCS	*Oryza sativa*	2004-8-1	DQ299168
103	SJN3.04	China	NCS	*Oryza sativa*	2004-8-1	DQ299169
104	SJN4.04	China	NCS	*Oryza sativa*	2004-8-1	DQ299170
105	LS.SDJN051	China	NCS	*Oryza sativa*	2005-4-30	EF198690
106	JN	China	NCS	*Oryza sativa*	1997	AF220105
107	LS.SDJN052	China	NCS	*Oryza sativa*	2005-4-30	EF198680
108	SJN07	China	NCS	*Oryza sativa*	2007-7-1	EU931512
109	HKF07	China	NCS	*Oryza sativa*	2007-7-1	EU931520
110	SQ	China	NCS	*Oryza sativa*	1997	AF220108
111	LS.HBTH05	China	NCS	*Oryza sativa*	2005-7-29	EF493227
112	HN.TH.03	China	NCS	*Oryza sativa*	2003-7-1	AY597383
113	BWQ1.05	China	NCS	*Oryza sativa*	2005-7-1	DQ299158
114	TXQ1.04	China	NCS	*Oryza sativa*	2004-7-1	DQ299171
115	TXQ2.04	China	NCS	*Oryza sativa*	2004-7-1	DQ299172
116	TXQ3.04	China	NCS	*Oryza sativa*	2004-7-1	DQ299173
117	TXQ4.04	China	NCS	*Oryza sativa*	2004-7-1	DQ299174
118	HXH3.05	China	NCS	*Oryza sativa*	2005-8-1	DQ299157
119	HXH1.05	China	NCS	*Oryza sativa*	2005-8-1	DQ299159
120	HXH2.05	China	NCS	*Oryza sativa*	2005-8-1	DQ299160
121	YY	China	NCS	*Oryza sativa*	2001	AY284859
122	YY.2002	China	NCS	*Oryza sativa*	2002-7-1	AY289050
123	HN.YY.03	China	NCS	*Oryza sativa*	2003-7-1	AY597386
124	HN.YY.04	China	NCS	*Oryza sativa*	2004	AY973312
125	HYY3.04	China	NCS	*Oryza sativa*	2004-8-1	DQ299164
126	HYY1.04	China	NCS	*Oryza sativa*	2004-8-1	DQ302795
127	HYY2.04	China	NCS	*Oryza sativa*	2004-8-1	DQ302796
128	HZD1.05	China	NCS	*Oryza sativa*	2005-8-1	DQ299161
129	HZD2.05	China	NCS	*Oryza sativa*	2005-8-1	DQ299162

130	HZD3.05	China	NCS	*Oryza sativa*	2005-8-1	DQ299163
131	HZD1.04	China	NCS	*Oryza sativa*	2004-8-1	DQ299165
132	HZD2.04	China	NCS	*Oryza sativa*	2004-8-1	DQ302797
133	HZZ1.04	China	NCS	*Oryza sativa*	2004-8-1	DQ299166
134	HN.ZM.03	China	NCS	*Oryza sativa*	2003-7-1	AY597387
135	HN.ZM.04	China	NCS	*Oryza sativa*	2004	AY973313
136	BS	China	SWM	*Oryza sativa*	1997	AF220103
137	BS1	China	SWM	*Oryza sativa*	2000	AY284874
138	BS2	China	SWM	*Oryza sativa*	2001	AY286099
139	BS.2002	China	SWM	*Oryza sativa*	2002-7-1	AY289039
140	YN.BS.03	China	SWM	*Oryza sativa*	2003-7-1	AY597375
141	LS.YNBS041	China	SWM	*Oryza sativa*	2004-7-16	EF198687
142	LS.YNBS042	China	SWM	*Oryza sativa*	2004-7-16	EF198698
143	YBS07	China	SWM	*Oryza sativa*	2007-7-1	EU931500
144	CX1	China	SWM	*Oryza sativa*	2001	AY284869
145	CX2	China	SWM	*Oryza sativa*	2001	AY284870
146	CX3	China	SWM	*Oryza sativa*	2001	AY284871
147	CX.2002	China	SWM	*Oryza sativa*	2002	AY289040
148	YN.CX.03	China	SWM	*Oryza sativa*	2003-7-1	AY597376
149	YN.CX.03E	China	SWM	*Oryza sativa*	2003-7-1	AY597377
150	YN.CX.03.M3	China	SWM	*Oryza sativa*	2003-7-1	AY597388
151	YN.CX.03.M4	China	SWM	*Oryza sativa*	2003-7-1	AY597389
152	YN.CX.03.M5	China	SWM	*Oryza sativa*	2003-7-1	AY597390
153	YN.CX.03.M9	China	SWM	*Oryza sativa*	2003-7-1	AY597391
154	YN.CX.02	China	SWM	*Oryza sativa*	2002-7-1	AY597392
155	YCX07	China	SWM	*Oryza sativa*	2007-7-1	EU931496
156	YCXi8	China	SWM	*Oryza sativa*	2013-7-3	KP083239
157	Chinese.Y	China	SWM	*Oryza sativa*	1995	Y11095
158	DL1	China	SWM	*Oryza sativa*	2000	AY284865
159	DL2	China	SWM	*Oryza sativa*	2001	AY284866
160	DL3	China	SWM	*Oryza sativa*	2001	AY284867
161	DL.2002	China	SWM	*Oryza sativa*	2002-7-1	AY289041
162	YN.DL.03	China	SWM	*Oryza sativa*	2003-7-1	AY597378
163	LS.YNDL04	China	SWM	*Oryza sativa*	2004-7-16	EF198683
164	FM1	China	SWM	*Oryza sativa*	2001	AY284861
165	FM2	China	SWM	*Oryza sativa*	2001	AY284862
166	HT.2002	China	SWM	*Oryza sativa*	2002-7-1	AY289042
167	JC	China	SWM	*Oryza sativa*	2001	AY284860
168	XS.2002	China	SWM	*Oryza sativa*	2002-7-1	AY289047
169	KM	China	SWM	*Oryza sativa*	2001	AF508912
170	KM	China	SWM	*Oryza sativa*	2000 or 2001	AY286106
171	YLFe10	China	SWM	*Oryza sativa*	2013-7-3	KP083234
172	YN.LL.03	China	SWM	*Oryza sativa*	2003-7-1	AY597380

173	LQ.2002	China	SWM	*Oryza sativa*	2002-7-1	AY289043
174	YN.LQ.03	China	SWM	*Oryza sativa*	2003-7-1	AY597381
175	YQJ07	China	SWM	*Oryza sativa*	2007-7-1	EU931504
176	YSDi28	China	SWM	*Oryza sativa*	2013-7-25	KP083235
177	YN.SL.03	China	SWM	*Oryza sativa*	2003-7-1	AY597382
178	Huid04	China	SWM	*Oryza sativa*	2004	KX611339
179	YSMi6	China	SWM	*Oryza sativa*	2013-7-30	KP083236
180	WS	China	SWM	*Oryza sativa*	2001	AY286104
181	WD	China	SWM	*Oryza sativa*	2001	AY286103
182	WD.2002	China	SWM	*Oryza sativa*	2002-7-1	AY289046
183	YN.WD.03	China	SWM	*Oryza sativa*	2003-7-1	AY597384
184	WD04	China	SWM	*Oryza sativa*	2004	AY973551
185	YYAn9	China	SWM	*Oryza sativa*	2013-7-3	KP083237
186	YL	China	SWM	*Oryza sativa*	1997	AF220109
187	YL	China	SWM	*Oryza sativa*	2001	AF508913
188	YL1	China	SWM	*Oryza sativa*	2001	AY284868
189	YL2	China	SWM	*Oryza sativa*	2001	AY286100
190	YR	China	SWM	*Oryza sativa*	2001	AY286102
191	YR.2002	China	SWM	*Oryza sativa*	2002-7-1	AY289048
192	DaL08	China	SWM	*Oryza sativa*	2008-7-1	JQ927420
193	KunM08	China	SWM	*Oryza sativa*	2008-7-1	JQ927423
194	BSh04	China	SWM	*Oryza sativa*	2004	AJ875061
195	DYa04	China	SWM	*Oryza sativa*	2004	AJ875062
196	YAn04	China	SWM	*Oryza sativa*	2004	AJ875060
197	FYi04	China	SWM	*Oryza sativa*	2004	AJ875058
198	FMi04	China	SWM	*Oryza sativa*	2004	AJ875057
199	LLi04	China	SWM	*Oryza sativa*	2004	AJ875059
200	BSh1	China	SWM	*Oryza sativa*	2003-8-1	AJ781025
201	CXi1	China	SWM	*Oryza sativa*	2003-8-1	AJ781026
202	SLi1	China	SWM	*Oryza sativa*	2003-8-1	AJ781027
203	YLi1	China	SWM	*Oryza sativa*	2003-8-1	AJ781028
204	YWS	China	SWM	*Oryza sativa*	2009	FM242703
205	YYL	China	SWM	*Oryza sativa*	2008	FM242704
206	YWD07	China	SWM	*Oryza sativa*	2007-7-1	EU931508
207	YX	China	SWM	*Oryza sativa*	2001	AY286107
208	YX.2002	China	SWM	*Oryza sativa*	2002-7-1	AY289049
209	YN.YX.03	China	SWM	*Oryza sativa*	2003-7-1	AY597385
