# Supplementary material for: Bayesian Phylogeographic Inference Suggests Japan as the Center for the Origin and Dissemination of Rice Stripe Virus
Source: Viruses. 2022 Nov 17;14(11):2547. doi: 10.3390/v14112547 (PMC9698939; doi:10.3390/v14112547)
Supplement: Supplementary file 1 [file viruses-14-02547-s001.zip › Table S2.pdf]

Table S2 Marginal likelihoods of different combinations of clock model and tree prior

Molecular model	Coalescent tree prior	Log marginal likelihood
Strict clock	Bayesian skyline	-9989.138
Strict clock	Constant size	-9990.471
**Strict clock**	**MASCOT**	**-9434.610**
Uncorrelated lognormal relaxed clock	Bayesian skyline	-10443.327
Uncorrelated lognormal relaxed clock	Constant size	-10560.719
Uncorrelated lognormal relaxed clock	MASCOT	-9711.672

The best-fitting tree prior and molecular clock model are indicated in bold font.

MASCOT, the marginal approximation of the structured coalescent
