# Supplementary figures and images for: Bayesian Phylogeographic Inference Suggests Japan as the Center for the Origin and Dissemination of Rice Stripe Virus
Source: Viruses. 2022 Nov 17;14(11):2547. doi: 10.3390/v14112547 (PMC9698939; doi:10.3390/v14112547)

Figure S1

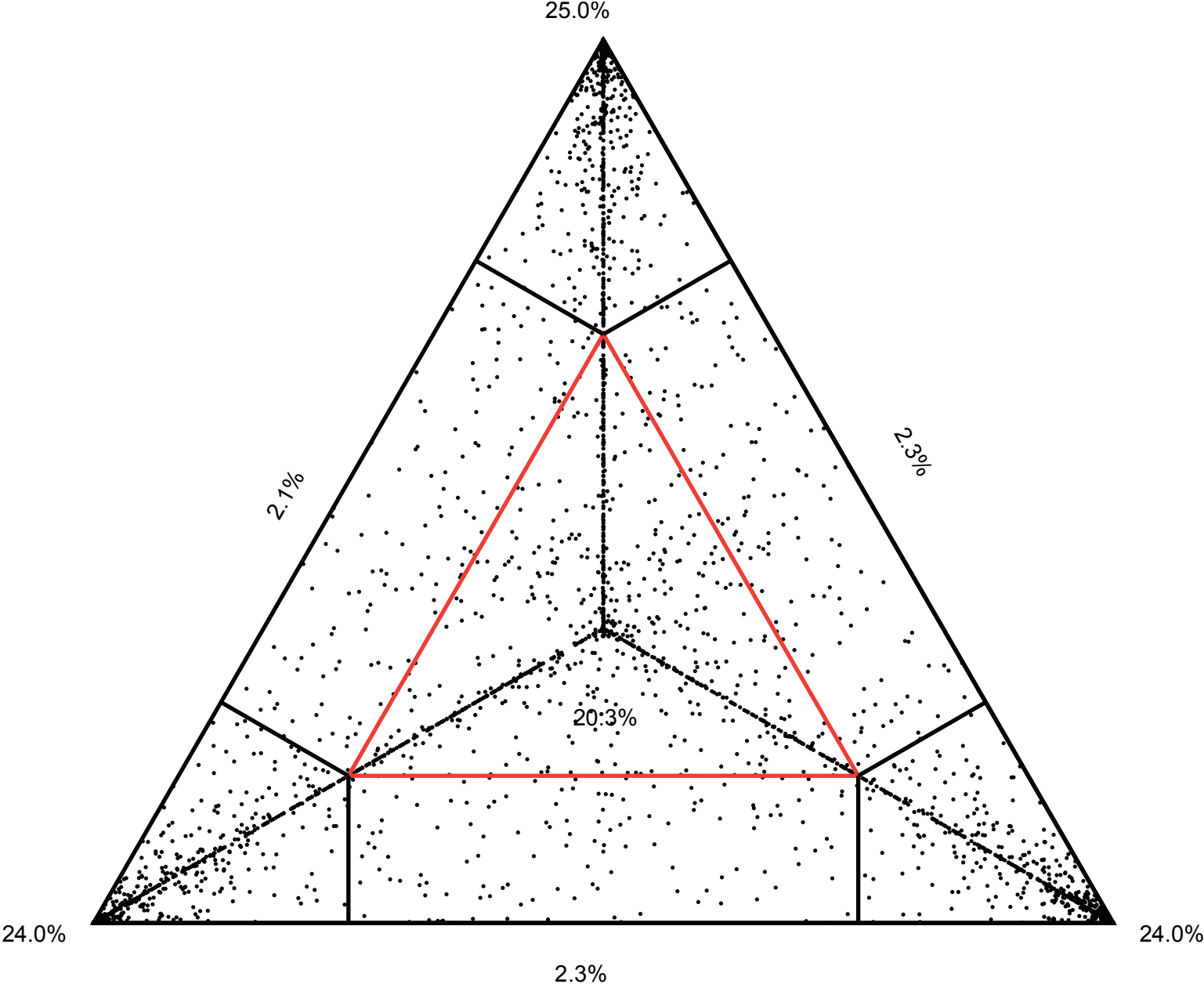

Supplement: Supplementary file 1 [file viruses-14-02547-s001.zip › Figure S1.pdf]

Figure S2

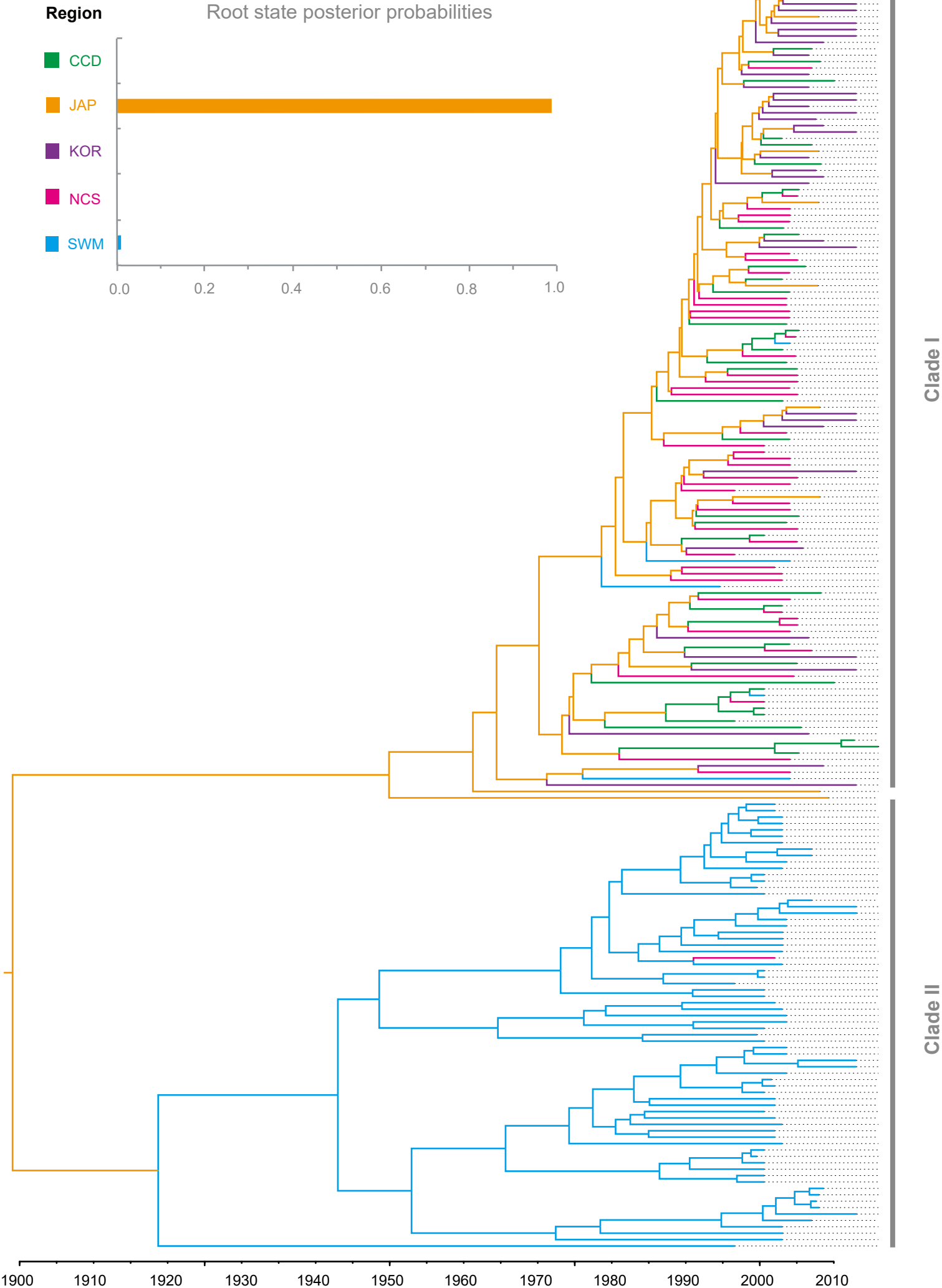

Supplement: Supplementary file 1 [file viruses-14-02547-s001.zip › Figure S2.pdf]

Figure S3

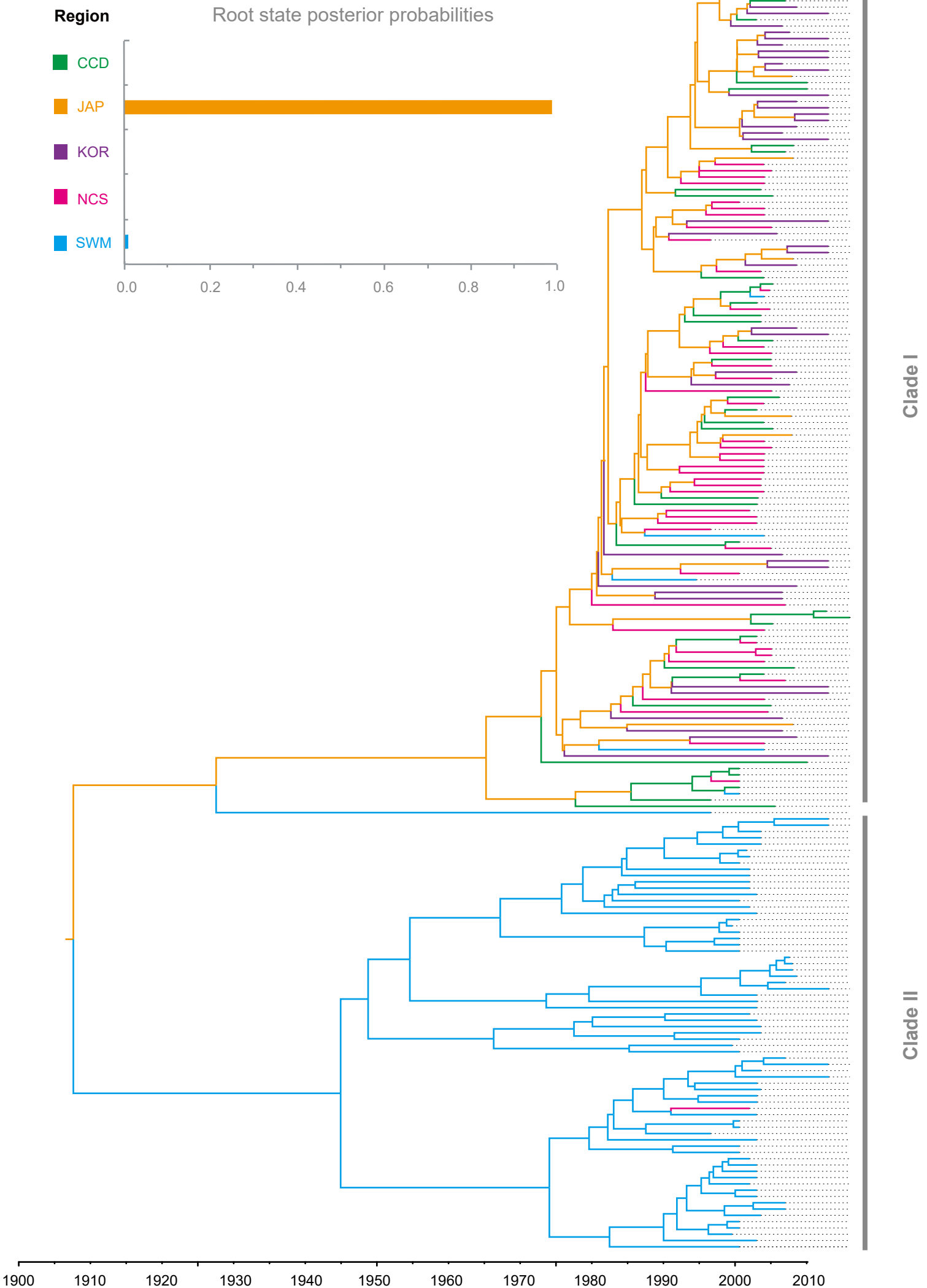

Supplement: Supplementary file 1 [file viruses-14-02547-s001.zip › Figure S3.pdf]

Figure S4

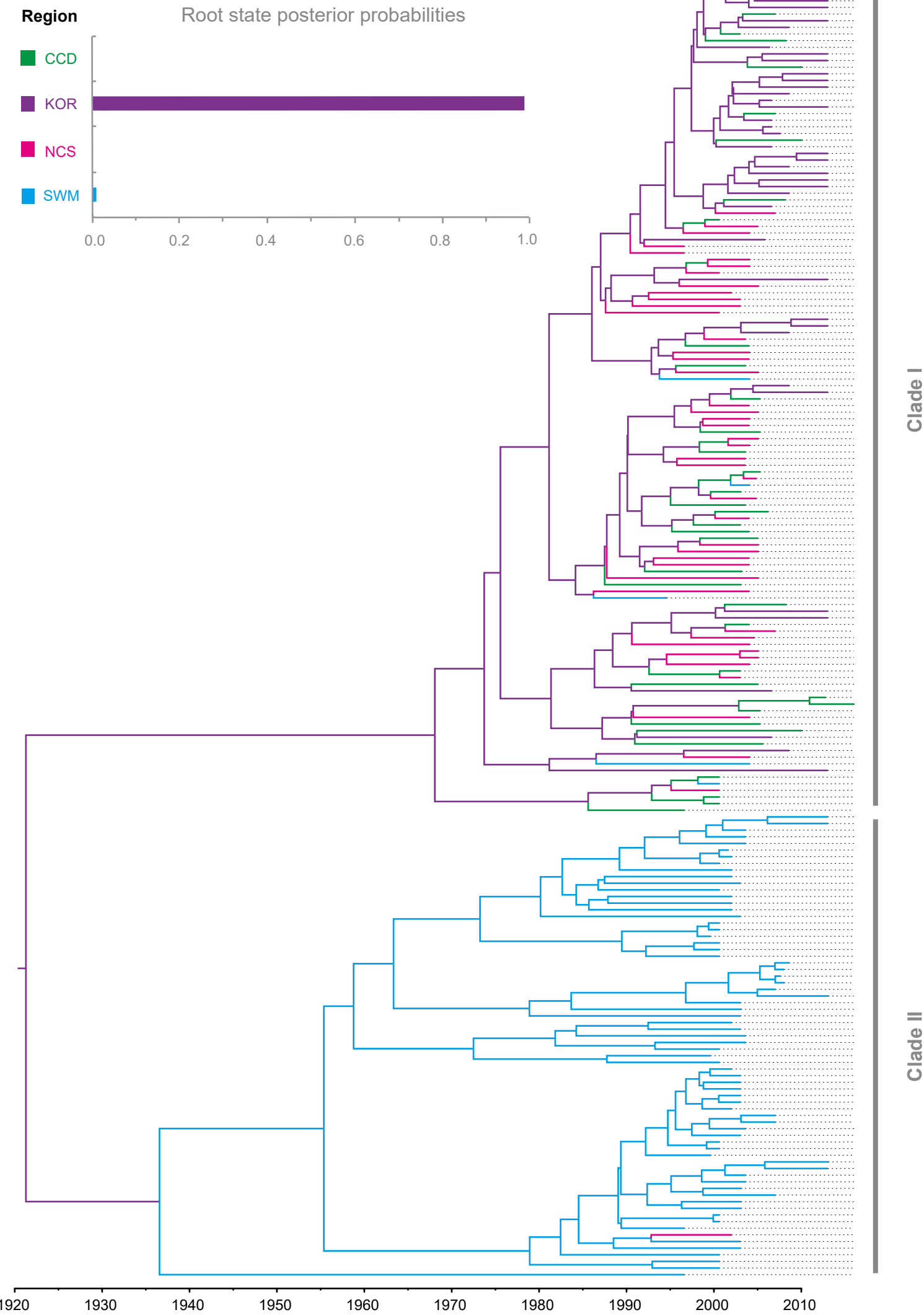

Supplement: Supplementary file 1 [file viruses-14-02547-s001.zip › Figure S4.pdf]

Figure S5

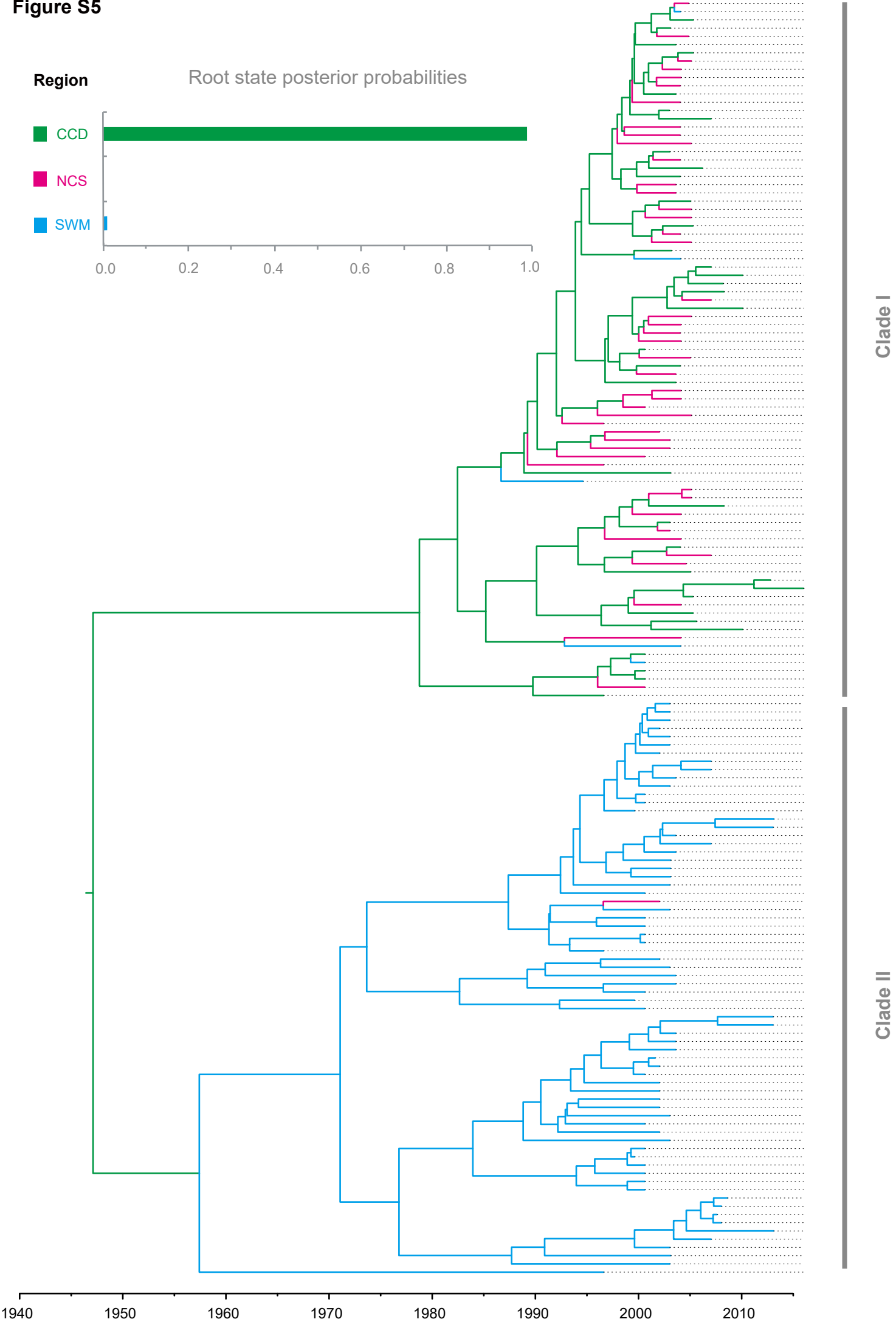

Supplement: Supplementary file 1 [file viruses-14-02547-s001.zip › Figure S5.pdf]
